# Prevalence and Risk Factors of Prostate Cancer in Chinese Men with PSA 4–10 ng/mL Who Underwent TRUS-Guided Prostate Biopsy: The Utilization of PAMD Score

**DOI:** 10.1155/2015/596797

**Published:** 2015-10-18

**Authors:** Dong Fang, Da Ren, Chenglin Zhao, Xuesong Li, Wei Yu, Rui Wang, Huihui Wang, Chenguang Xi, Qun He, Xiaoying Wang, Zhongcheng Xin, Liqun Zhou

**Affiliations:** ^1^Department of Urology, Peking University First Hospital, Institute of Urology, Peking University, National Urological Cancer Center, No. 8 Xishiku Street, Xicheng District, Beijing 100034, China; ^2^Department of Radiology, Peking University First Hospital, No. 8 Xishiku Street, Xicheng District, Beijing 100034, China

## Abstract

*Purpose*. To elucidate the characteristics and risk factors for positive biopsy outcomes in Chinese patients with prostate specific antigen (PSA) 4–10 ng/mL and develop a risk-stratification score model. *Methods*. The data of 345 patients who underwent transrectal ultrasound-guided prostate biopsy between 2011 and 2013 was retrospectively analyzed. Digital rectal examination (DRE), prostate volume (PV), magnetic resonance imaging (MRI), and smoking status were also collected. Positive biopsy outcomes were defined as prostate cancer (PCa) and high grade PCa (HGPCa, Gleason Score ≥ 7). *Results*. The median PSA was 7.15 (IQR 5.91–8.45) ng/mL. Overall 138 patients (40.0%) were shown to have PCa, including 100 patients (29.0%) with HGPCa. Smaller PV, elder age, MRI results, and positive DRE were proved to be predictive factors for positive biopsy outcomes in both univariate and multivariate analysis. We developed a “PAMD” score which combined the four factors to categorize patients into three risk groups, and the model performed good predictive sensitivity and specificity. *Conclusion*. The prevalence of prostate cancer in Chinese patients with PSA 4–10 ng/mL was 40%, including 29% patients with high grade disease. DRE, age, MRI, and PV were predictive factors for positive biopsy outcomes, and the PAMD score model could be utilized for risk-stratification and decision-making.

## 1. Introduction 

Systematic transrectal ultrasound- (TRUS-) guided prostate biopsy is currently the standard practice for the diagnosis of prostate cancer (PCa) [1]. According to European Association of Urology (EAU) guidelines, the decision to undergo biopsy should be based on prostate specific antigen (PSA) and digital rectal examination (DRE) [1]. There has been significant development in the use of multiparametric-magnetic resonance imaging (MRI) in diagnosis of prostate cancer [2–4] in recent years, but whether MRI could act as a useful evaluating tool for the decision for biopsy remains unclear [5].

As the most commonly used screening tool, the elevated value of PSA often indicated the need for prostate biopsy. Traditionally 4 ng/mL was often regarded as the cut-off to consider biopsy [6], but controversy exists about the biopsy indication for patients with PSA between 4 and 10 ng/mL [7–9]. The positive rate ranges variously in previous reports (20.6%–39.1%) and PSA alone has low predictive accuracy [10].

Besides, there are few prostate cancer screening protocols in China, and the epidemiologic characteristics in this unique population are unknown [11–14]. It is essential to elucidate the prevalence of PCa in various PSA range, especially in the “gray zone,” to provide more optimal and personalized risk-based therapy options, and further achieve better disease management for patients with elevated PSA in China.

Thus based on this large cohort of Chinese patients, we sought to elucidate the characteristics and risk factors for positive biopsy outcomes and develop a risk-stratification score model for clinical use.

## 2. Materials and Methods

### 2.1. Patient Selection

Following institutional review board approval and written informed consent from patients, we initially collected information of 1348 patients who underwent TRUS-guided prostate biopsy between January 2011 and November 2013. All patients were referred for a prostate evaluation because of elevated PSA and/or suspicious DRE or TRUS. We exclude patients who received previous prostate biopsy or neoadjuvant hormonal therapy before biopsy and patients with incomplete data or extreme values (e.g., prostate volume > 150 mL). Three hundred and forty-five patients with prebiopsy PSA between 4 ng/mL and 10 ng/mL were finally enrolled for evaluation. Eligible patients were unselected and were accrued consecutively.

### 2.2. Patients Evaluation

PSA levels including the percentage of free PSA (%fPSA) were measured before DRE and TRUS. DRE result was defined as unsuspicious versus suspicious. Prostate volume (PV) was determined by TRUS and was calculated using the formula (width × length × height × 0.52). By two experienced doctors, TRUS-guided systematic biopsies of ≥10 cores (usually 12 or 13 needles) plus targeted biopsies at any suspicious area for malignancy were performed in all patients. All biopsy specimens were evaluated by a dedicated genitourinary pathologist to determine the presence of PCa and the Gleason score in positive cases. High grade PCa (HGPCa) was defined as Gleason score ≥7. Because of the extremely low prevalence in Chinese population, family history was not analyzed. Positive smoking history was defined as over 10 years' duration of smoking with over 20 cigarettes per day.

MRI studies were performed at 1.5T or 3T; all MRI studies involved the sequences and acquisition parameters of axial T1-weighted imaging, axial and coronal T2-weighted fast spin-echo imaging, axial diffusion weighted imaging, and axial dynamic contrast enhanced imaging. Two experienced radiologists retrospectively and independently interpreted the MR images. Any disagreement was resolved by the adjudicating senior radiologist. The MRI diagnosis was evaluated by using a three-point scale in consideration of clinical use and the PI-RADS criteria [15]: Grade 0 (similar to PI-RADS 1-2), clinically significant disease is highly unlikely to be present; Grade 1 (similar to PI-RADS 3), clinically significant cancer is equivocal or suspicious; Grade 2 (similar to PI-RADS 4-5), clinically significant cancer is highly likely to be present.

### 2.3. Statistical Analysis

Pearson's test and Chi-square test were used to test the distribution of categorical variables, and the Mann-Whitney *U* test was used for continuous variables. Multivariate logistic regression was used to calculate the predictive factors. Multivariate logistic regression coefficients were used to generate a risk-stratification score. Receiver operating characteristic (ROC) curves were generated to illustrate the predictive accuracy. All statistical tests were performed with SPSS 20.0 (IBM Corp., Armonk, NY, USA). All reported *p* values were two-sided with statistical significance considered at *p* < 0.05.

## 3. Results and Discussion

### 3.1. Patients Demographics

**Figure 1:**
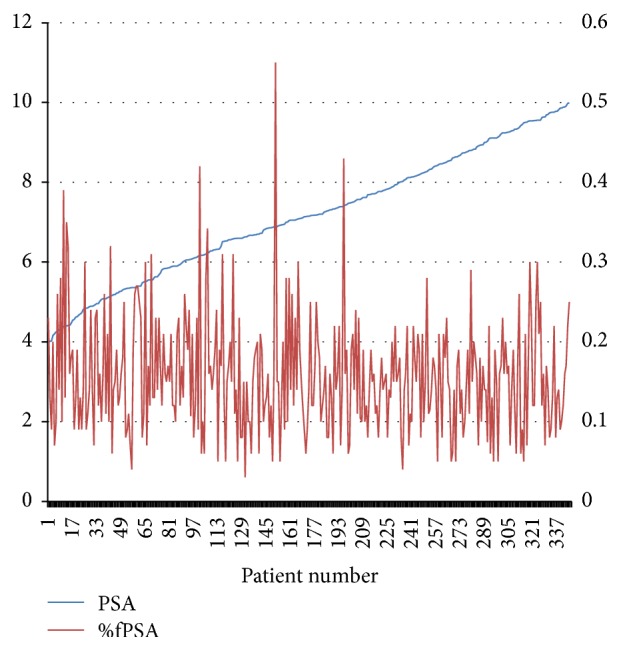
Distribution of PSA and %fPSA. PSA = prostate specific antigen; %fPSA = the percentage of free PSA.

The median age of this cohort of patients was 67 (interquartile range, IQR 61–74) years. The median PSA was 7.15 (IQR 5.91–8.45) ng/mL, and the median %fPSA was 0.15 (IQR 0.10–0.19) (Figure 1). The median PV was 50 (IQR 36.7–69.9) mL. Negative and positive DRE were present in 292 (84.6%) and 53 (15.4%) patients each. For MRI grades, Grade 0 (negative) was assigned in 132 patients (38.3%), while Grade 1 (suspicious) and Grade 2 (positive) were assigned in 79 patients (22.9%) and 134 patients (38.8%), respectively. Considering the low diagnostic accuracy and different criteria of TRUS, we did not include the result of TRUS as a risk factor.

### 3.2. Univariate and Multivariate Analysis of Risk Factors for Positive Biopsy Results

Overall 138 patients (40.0%) were shown to have PCa, and particularly 100 patients (29.0%) were diagnosed with HGPCa.

**Figure 2:**
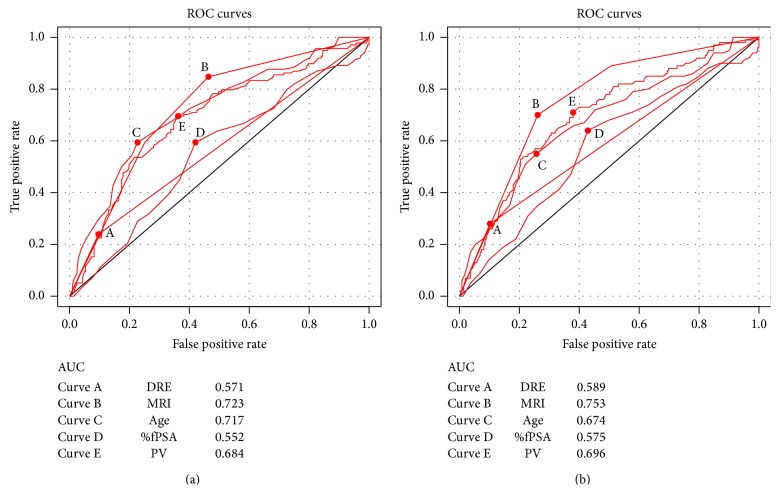
ROC curves of different risk factors in predicting PCa (a) and HGPCa (b). ROC = receiver operating characteristic; DRE = digital rectal examination; MRI = magnetic resonance imaging; %fPSA = the percentage of free PSA; PV = prostate volume; AUC = area under the receiver operating characteristics curve.

In univariate analysis, elder age, lower %fPSA, suspicious DRE, MRI grades, and smaller PV were related to the presence of PCa and HGPCa (Table 1). It is notable that in ROC curves fPSA exhibited poor ability in predicting the presence of PCa (area under the receiver operating characteristics curve, AUC = 0.552) and HGPCa (AUC = 0.575) (Figure 2). The highest AUC for a single risk factor is MRI (AUC = 0.723 for PCa and AUC = 0.753 for HGPCa).

PV, age, MRI grades, and DRE remained independent predictor in multivariate analysis in predicting both PCa and HGPCa, while %fPSA exhibited no statistical correlation when controlling for other factors (Table 2).

### 3.3. Construction of Risk-Stratification Model

According to the multivariate relative risk, we proposed a risk factor-based stratification model: a “PAMD” score. Each score was defined as the approximate value of their coefficient. Finally each risk factor was scored as follows: PV > 50 mL = 0 points, PV≦50 mL = 2 points; age ≤ 68 = 0 points, age > 68 = 2 points; negative MRI = 0 points, suspicious MRI = 1 point, and positive MRI = 2 points; negative DRE = 0 points, positive DRE = 1 point. PAMD score was defined as the sum of the scores, which represented the combination of the four independent risk factors (PV, age, MRI, and DRE).

**Figure 3:**
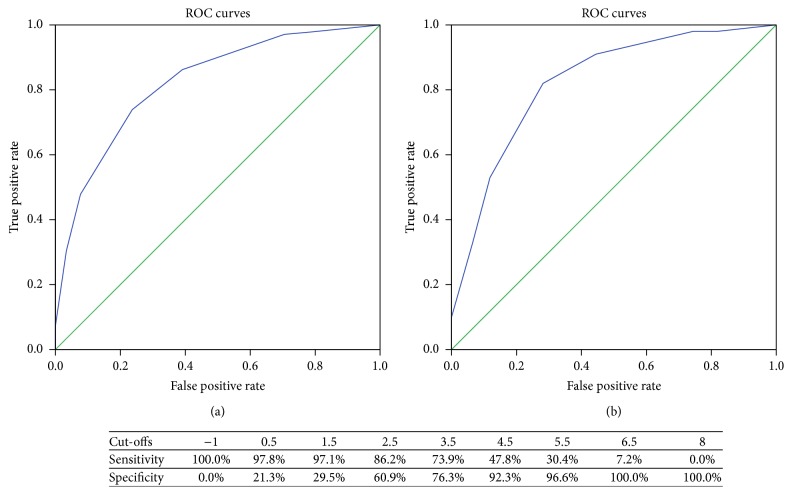
ROC curves of PAMD score in predicting PCa (a) and HGPCa (b). ROC = receiver operating characteristic; PAMD = prostate volume + age + magnetic resonance imaging + digital rectal examination. Note that the table presents various cut-offs and the corresponding sensitivities and specificities in predicting PCa.

As is shown in Figure 3, the PAMD score had high predictive accuracy for PCa (AUC = 0.822) and HGPCa (AUC = 0.824). For the convenience of clinical practice, various cut-offs and the corresponding sensitivities and specificities (in predicting PCa) of the model were also listed in Figure 3. For patients with only PAMD score <2, the low specificity which indicates the low possibility of prostate cancer could remind clinicians to reevaluate the necessity of biopsy.

All 345 patients could be divided into three risk groups according to the PAMD score: low (0-1 point), intermediate (2-3 points), and high (over 4 points). There is significant difference in biopsy outcomes between the three risk groups with *p* < 0.001 (Table 3).

### 3.4. Discussion

The commonly regarded cut-off value of PSA to consider biopsy is 4 ng/mL [6], but to perform biopsy to all patients beyond that criteria would result in many unnecessary examinations and overdiagnosis for insignificant disease [16, 17], and the great economic burden makes it difficult in developing countries. Thus for patients without an extremely high value of PSA (e.g., PSA > 50 ng/mL), an easy risk-stratification method which would bring benefits in terms of costs and patient satisfaction is required.

For patients with PSA 4–10 ng/mL, several articles have focused on risk factors for biopsy outcomes [7–9], and most of these studies were based on developed countries (America, Japan, and Korea). Our study confirmed that DRE, age, MRI, and PV were independent predictors for PCa. It is interesting that there is no difference in PSA value between PCa and noncancer patients; we believe the higher the PSA value, the more likely the existence of PCa; probably sample size and selection bias affected this analysis. We did not include the result of TRUS because there is no clear consensus about the criteria [18, 19], and individual opinion of various doctors would bias the result.

The prevalence of PCa in this cohort of Chinese patients was 40%, including 29% high grade disease, which indicates clinicians should pay attention to the risk for PCa in this PSA range. The sensitivity and specificity at different cut-offs of PAMD score could help decision-making. It is notable that patients with PAMD score over 2 points (intermediate or high risk) would have at least 24.8% possibility to have PCa and 12.4% possibility to have HGPCa. Clinicians should fully consider the option for biopsy even if for patients with only 1 risk factor (e.g., a positive MRI). For low-risk patients, the decision to undergo biopsy could be based on clinical judgment of treating physician in consideration of clinical characteristics and thorough discussion with patient about possible options and expectations.

The %fPSA was recommended in many previous papers which exhibit good predictive accuracy [7–10, 20, 21]. The EAU guideline recommends %fPSA to be routinely considered in every patient with suspicious findings [1]; for Chinese patients, the current Chinese Urological Association (CUA) guideline also proposed that patients with %fPSA > 0.16 and PSA 4–10 ng/mL should be referred to biopsy [22]. In the present study, %fPSA had unsatisfied predictive sensitivity and specificity in univariate analysis and showed no statistical significance in multivariate analysis. Probably the incorporation of other factors, including MRI and PV, weakened the impact of %fPSA. Considering the disparity of epidemiology and biology of PCa between Western and Asian men [10, 23], the significance of %fPSA in this unique population might not be that strong as was reported.

In current guidelines there is no recommendation for the use of MRI for early detection [1, 24, 25]. There are just a few published articles evaluating whether MRI prior to initial biopsy contributed to the detection of prostate cancer [2, 26–28]. The present study supported the early detective role of MRI in this cohort of patients, which indicates that MRI result could be helpful in decision for biopsy. This is the first study to demonstrate the importance to include the MRI information into prebiopsy models. Although MR-fusion and MR-guided biopsy have gained significant development these years [29, 30], in the era that TRUS-guided systematic biopsy is still the prevalent biopsy protocol, a prebiopsy MRI could assist in disease control.

The limitations of this study include the retrospective design and data collection, which did not allow us to evaluate some potentially useful variables such as PSA-velocity, PCA3, treatment outcomes, and survival information; and our study cohort might be subject to selection and recall bias. The relatively small study sample size might have affected the analysis and further external validation is required. Future prospective multicenter studies and maybe some screening trials would be required to fully elucidate the prevalence of PCa in Chinese population.

## 4. Conclusions

The prevalence of prostate cancer in Chinese patients with PSA 4 ng/mL–10 ng/mL was 40%, including 29% patients with high grade disease. DRE, age, MRI, and PV were predictive factors for positive biopsy outcomes, and the PAMD score model could be utilized for risk-stratification and decision-making.

## Figures and Tables

**Table 1:** Baseline clinical characteristics and univariable analysis of risk factors for presence of PCa and HGPCa.

	All	Prostate cancer				High grade prostate cancer			
		No	Yes	Chi-square or *Z*	*p* value	No	Yes	Chi-square or *Z*	*p* value
Patients, number (%)	345 (100)	207 (60.0)	138 (40.0)			245 (71.0)	100 (29.0)		
PSA, mean ± SD		7.15 ± 1.57	7.16 ± 1.57	−0.126	0.900	7.13 ± 1.57	7.23 ± 1.57	−0.645	0.519
PSA, number (%)				0.124	0.742			0.540	0.477
≦7 nL/mL	159 (46.1)	97 (46.9)	62 (44.9)			116 (47.4)	43 (43.0)		
>7 ng/mL	186 (53.9)	110 (53.1)	76 (55.1)			129 (52.6)	57 (57.0)		
%fPSA, mean ± SD		0.158 ± 0.065	0.154 ± 0.081	−1.637	0.102	0.159 ± 0.065	0.152 ± 0.087	−2.191	0.028^*∗*^
%fPSA, number (%)				7.029	0.005^*∗*^			9.511	0.001^*∗*^
<0.16	190 (55.1)	102 (49.3)	88 (63.8)			122 (49.8)	68 (68.0)		
≧0.16	155 (44.9)	105 (50.7)	50 (36.2)			123 (50.2)	32 (32.0)		
PV, mean ± SD		61.89 ± 26.37	47.78 ± 24.07	−5.783	**<**0.001^*∗*^	60.82 ± 26.85	45.04 ± 21.46	−5.711	**<**0.001^*∗*^
PV, number (%)				32.664	**<**0.001^*∗*^			27.955	**<**0.001^*∗*^
≦50 mL	175 (50.7)	79 (38.2)	96 (69.6)			102 (41.6)	73 (73.0)		
>50 mL	170 (49.3)	128 (61.8)	42 (30.4)			143 (58.4)	27 (27.0)		
Age, mean ± SD		64.06 ± 9.10	70.74 ± 7.52	−6.836	**<**0.001^*∗*^	65.16 ± 9.06	70.60 ± 8.03	−5.066	**<**0.001^*∗*^
Age, number (%)				38.074	**<**0.001^*∗*^			21.804	**<**0.001^*∗*^
≤68	185 (53.6)	139 (67.1)	46 (33.3)			151 (61.6)	34 (34.0)		
>68	160 (46.4)	68 (32.9)	92 (66.7)			94 (38.4)	66 (66.0)		
MRI, number (%)				57.610	**<**0.001^*∗*^			63.486	**<**0.001^*∗*^
Negative	132 (38.3)	111 (53.6)	21 (15.2)			121 (49.4)	11 (11.0)		
Suspicious	79 (22.9)	44 (21.3)	35 (25.4)			60 (24.5)	19 (19.0)		
Positive	134 (38.8)	52 (25.1)	82 (59.4)			64 (26.1)	70 (70.0)		
DRE, number (%)				12.933	**<**0.001^*∗*^			17.297	**<**0.001^*∗*^
Unsuspicious	292 (84.6)	187 (90.3)	105 (76.1)			220 (89.8)	72 (72.0)		
Suspicious	53 (15.4)	20 (9.7)	33 (23.9)			25 (10.2)	28 (28.0)		
Smoke, number (%)				0.004	0.953			0.236	0.627
Absent	288 (83.5)	173 (83.6)	115 (83.3)			203 (82.9)	85 (85.0)		
Present	57 (16.5)	34 (16.4)	23 (16.7)			42 (17.1)	15 (15.0)		

^*∗*^Statistically significant.

PCa = prostate cancer; HGPCa = high grade prostate cancer; PSA = prostate specific antigen; %fPSA = percentage of free PSA; PV = prostate volume; MRI = magnetic resonance imaging; DRE = digital rectal examination.

**Table 2:** Multivariable analysis of risk factors for presence of PCa and HGPCa.

	Prostate cancer				High grade prostate cancer			
	Coefficient	OR	95% CI	*p* value	Coefficient	OR	95% CI	*p* value
%fPSA^∧^	−0.357	0.700	0.391–1.252	0.229	−0.491	0.612	0.331–1.133	0.118
PV^∧^	1.555	4.736	2.687–8.348	**<**0.001^*∗*^	1.407	4.082	2.237–7.451	**<**0.001^*∗*^
Age^∧^	1.513	4.539	2.591–7.952	**<**0.001^*∗*^	1.017	2.765	1.546–4.948	0.001^*∗*^
MRI	1.607	4.990	2.687–9.266	**<**0.001^*∗*^	2.103	8.187	3.970–16.884	**<**0.001^*∗*^
DRE	0.861	2.366	1.162–4.815	0.018^*∗*^	1.108	3.027	1.479–6.194	0.002^*∗*^

^*∗*^Statistically significant.

^∧^Calculated as dichotomous variable (%fPSA ≤0.16 versus >0.16; PV ≦50 mL versus >50 mL; age ≤68 versus >68).

PCa = prostate cancer; HGPCa = high grade prostate cancer; %fPSA = percentage of free PSA; PV = prostate volume; MRI = magnetic resonance imaging; DRE = digital rectal examination; OR = Odds Ratio; CI = confidence interval.

**Table 3:** Biopsy outcomes between different risk groups stratified by PAMD score.

	PAMD score	ALL	PCa				HGPCa			
			No	Yes	Chi-square	*p*	No	Yes	Chi-square	*p*
Low	0-1	65	61 (93.8)	4 (6.2)	91.187	**<**0.001^*∗*^	63 (96.9)	2 (3.1)	85.457	**<**0.001^*∗*^
Intermediate	2-3	129	97 (75.2)	32 (24.8)			113 (87.6)	16 (12.4%)		
High	Over 4	151	49 (32.4)	102 (67.6)			69 (45.7)	82 (54.3)		

^*∗*^Statistically significant.

PAMD = prostate volume + age + magnetic resonance imaging + digital rectal examination; PCa = prostate cancer; HGPCa = high grade prostate cancer.
